# Supplementary material for: Comparative multiomics analysis of cell physiological state after culture in a basket bioreactor
Source: Sci Rep. 2022 Nov 23;12:20161. doi: 10.1038/s41598-022-24687-4 (PMC9686226; doi:10.1038/s41598-022-24687-4)
Supplement: Supplementary file 1 — Supplementary Information 1. [file 41598_2022_24687_MOESM1_ESM.zip › raw data/Proteomic raw data/1.data_control/cov_prot.pdf]

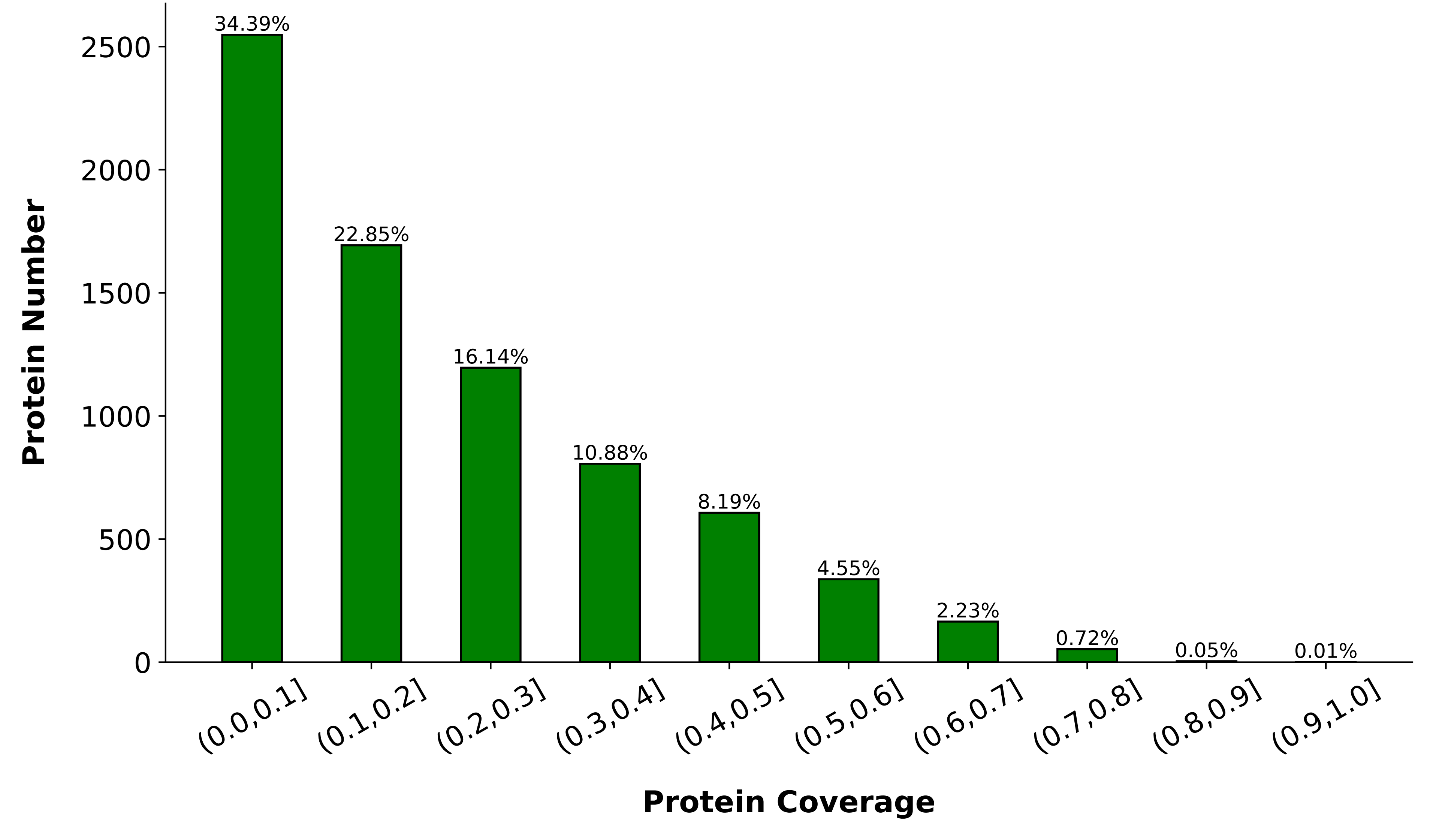

Protein Number
2500
2000
1500
1000
500
0
34.39%
22.85%
16.14%
10.88%
8.19%
4.55%
2.23%
0.72%
0.05%
0.01%
(0.0,0.1]
(0.1,0.2]
(0.2,0.3]
(0.3,0.4]
(0.4,0.5]
(0.5,0.6]
(0.6,0.7]
(0.7,0.8]
(0.8,0.9]
(0.9,1.0]
Protein Coverage
